# Prevalence of Depression Among Community Dwelling Elderly in Karachi, Pakistan 

**Published:** 2012

**Authors:** Seyed Muhammed Mubeen, Danish Henry, Sarah Nazimuddin Qureshi

**Affiliations:** 1 Hamdard College of Medicine Dentistry, Hamdard University, Karachi, Pakistan.

**Keywords:** Aged, Depression, Geriatrics

## Abstract

**Objective: **The objectives of the study were to find out the prevalence of depression and to identify associated risk factors among community dwelling elderly in Karachi.

**Methods: **It was a cross-sectional, descriptive study involving 284 community-dwelling elderly residing in Karachi, Pakistan. A non-probability convenience sampling was done. The Geriatric Depression Scale (GDS-15) was used to assess depression. Descriptive statistics was performed using SPSS version 12. Cross tabulation for different variables was done and Chi-square was used as test of significance. The level of significance was set as p < 0.05. An informal (verbal) consent was taken. Anonymity and confidentiality was assured.

**Results: **Among 284 respondents, 74% were males while 26% were females. The mean age was 68.44 ±7.59 years. The study found that 16.5% respondents were depressed while 23.6% were suggestive of depression. Depression was more among men than in women. Depression was statistically significant among married respondents (p<0.05) and illiterate (p<0.001). Although a large proportion of the participants were satisfied with their income, this was statistically significant (p<0.001) for depression among those who were not satisfied with their income. Similarly, sleep was significantly disturbed (p<0.001) among the depressed respondents.

**Conclusion: **A significant prevalence of geriatric depression was reported. In order to reduce its prevalence, general physicians and other health care professionals need to be sensitized about geriatric depression and its risk factors**.**

## Introduction

Depression is a common psychiatric disorder and the most common in geriatrics (1). Currently, depression is the 2^nd^ leading cause of Disability Adjusted Life Years (DALYs) in the age category 15-44 years for both sexes combined and by the year 2020, it is projected to reach the 2^nd^ place of the ranking of DALYs, calculated for all ages, both sexes (2).

Various studies have been conducted to investigate depression in the elderly and many found depression to be largely under diagnosed and untreated (3).Primary-care doctors rarely diagnose depression and, when they do provide inappropriate treatment (4). Barriers to adequate diagnosis and treatment include doctors’ reluctance to discuss emotional problems, time constraints and medical co-morbidities, complicating diagnosis and competing for medical attention. Perceived stigma contributes to patients’ reluctance to initiate psychiatric treatment (5). While late-life depression tends to be a recurring disorder, it may be masked by hypochondriasis or somatization (6). Furthermore, complex medication regimens undermine treatment adherence. It is not surprising that older patients with depressive disorder have high rates of treatment resistance and mortality (6,7).

In this globalized world, the ‘Empty Nest Syndrome’ is not just a Western phenomena; the younger generation has allowed it to make in-roads into the eastern culture. In Pakistani social setting, many people work past their retirement age (60 years in Pakistan) mainly due to compelling circumstances to make both ends meet. Also, the gradual disintegration of the centuries old joint family system, emergence of nuclear family system and individuality – self centeredness has eroded the channels of care for the elderly and has banished them to isolation with impunity (8).

Contributing factors include a fragile health system with no dedicated geriatric clinics, deteriorating social and moral fiber add to miseries of the elderly with poor checks and balances in pensions and old-age benefits, mechanical and fast way of life in the urban areas. It is coupled with intolerance, lack of harmony with nature, rat race for money and power, ruthlessness, disposable culture and rampant consumerism, looming insecurity, social injustice all are likely to drag a person into the gallows of depression, sometimes never to come back. Being denied what they have earned throughout their lives, these senior citizens become more prone to depression. 

The unattended depression is further aggravated by health care professional’s inability to recognize the importance of mental health. A study done in Lahore, Pakistan revealed that 50% of the medical students and professionals claimed that they had not heard about depression and a significant proportion of all the people surveyed had a negative attitude towards depressed patients (5). The objectives of the study were to find out the prevalence of depression among community-dwelling elderly in Karachi.

## Materials and Methods

It was a cross-sectional, descriptive study using non-probability convenience sampling method spanned over a period of 12 months (March 2009 to February 2010). The sample included community-dwelling elderly population residing in one of the four districts of Karachi, Pakistan. As there is no general agreement on the age at which a person becomes old, most of the developed world countries have accepted the chronological age of 65 years as a definition of 'elderly' or older person. Moreover, there is no UN standard numerical criterion, but the UN agreed cut-off is >60 years to refer to the older population (9). Considering the average lifetime expectancy of 67 years among a Pakistani population (10), elderly in this study refers to persons of age 60 years and above. With an estimated prevalence of 25% reported in pervious studies with 95% level of confidence and an absolute precision of 0.05, a sample size of 289 was calculated. Nonetheless, 300 persons were included in the study. Questionnaire of sixteen respondents were not included due to incomplete information, hence, 284 respondents were included as the final sample size. Elderly persons regularly visiting parks in afternoons and evenings and attending Masjid and church (places of worship) were invited to participate in the study. People going independently to these places are likely to have attentive cognition and can thus are able to sensibly respond to the survey questionnaire.

The questionnaire used in the study was divided into socio-demographic information and the GDS-15. The socio-demographic information included sex, age, residence, religion, mother tongue, education, marital status, occupation and family setup. It comprises Joint Family System (JFS) having two or more nuclear families that form a corporate economic unit (11) and Nuclear Family System (NFS) consisting of parent and their dependent children (11).Co-morbidities, use of daily medications and addiction(s) were also inquired.

The second part comprised of a scale for assessing late-life depression. It refers to depressive syndromes defined in the DSM-IV and in the ICD-10 that arise in adults older than age 65 years (3). For this assessment, The GDS-15, the shorter version of the questionnaire was selected. The usage of GDS-15 can be attributed to its easy administration to the target population and concise content, taking 5 to 7 minutes to complete. The scale can be used in healthy, medically ill and mild to moderate cognitively impaired older adults (12).This scale has been used both as a self-administered questionnaire and as read out interviews.

The GDS-15 assesses depression in the elderly on a score of 15. Scores of 0-4 are considered normal, depending on age, education, and complaints; 5-8 indicate mild depression; 9-11 signifies moderate depression and 12-15 specifies severe depression. Any positive score above 5 on the GDS-15 should prompt an in-depth psychological assessment and evaluation for sociality (12).

The GDS was found to have 92% sensitivity and 89% specificity when evaluated against diagnostic criteria. The validity and reliability of the tool have been supported through both clinical practice and research (13,14).Validation studies of the GDS-15 established it as a promising screening device for detecting depression in the elderly. An Australian study found GDS-15 to be a good screening instrument for major depression as defined both by ICD-10 and DSM-IV (15). Similarly, GDS with 10 and 15 items produced consistent results in the assessment of elderly patients when total scores were used as clinical guidelines (15). Nevertheless, GDS does not access suicidality and is not a substitute for a diagnostic interview by a mental health professional (12). 

The inclusion criteria were consenting individuals above the age of 60 years irrespective of sex, ethnicity or religion. Elderly suffering from chronic diseases like diabetes and hypertension were also included. Individuals below the age of 60, elderly suffering from known dementia and institutionalised elderly (in hospitals, old age homes, etc.) were excluded from the study. 

Statistical Package for Social Sciences (SPSS - V12) was used for statistical analysis and data management. The data was serialized for facilitating computer entries. Descriptive statistics was performed. Cross tabulation for different variables was done and Chi-square was used as test of significance to find out the association between two variables. The level of significance was set as p less than 0.05. 

The respondents of the questionnaire were informed of the purpose of the study and the usage of the information they provided. An informal (verbal) consent was taken before the respective respondents filled the questionnaire. The researcher(s) read out the questions for the respondents unable to read and write and recorded the responses. Anonymity and confidentiality of their information was assured and the right to withdraw upheld. The Research Committee of Hamdard University, Karachi gave the ethical approval.

## Results

Among 284 respondents, males (74%) were in a numerical majority than females (26%) and nearly half (47%) of the respondents were in the age bracket of 60 to 65 years. The mean age was 68.44 ±7.59 years. The majorities were Muslims (90%), married (77%) and spoke Urdu (72.4%) language. A little more than a quarter was illiterate and 7% of the respondents were unemployed at the time of study. Sixty-two percent were living as joint families while 47% of the respondents lived with their children only. Self- income was generated by 114 (40%) of the respondents whereas 55 (19%) rely on pension only. However, the majority (71%) were found to be satisfied with their income (Table 1).

**Table 1:** Demographic profile of the community dwelling elderly in Karachi Pakistan

	N	%
**Sex** Male Female	210 74	73.9 26.1
**Age (in years)** 60 – 65 66 – 70 71 – 75 76 – 80 > 80	132 69 39 28 16	46.5 24.3 13.7 9.9 5.6
**Marital status** Single Married Widow Separated / Divorced	12 218 43 11	4.2 76.8 15.1 3.9
**Religion ** Islam Hinduism Christianity	25.6 19 9	90.1 6.7 3.2
**Educational status** Primary Secondary Intermediate Graduate/diploma Postgraduate Illiterate	47 67 39 32 19 80	16.5 23.6 13.7 11.3 6.7 28.2
**Occupational status** Employed / self-employed Retired Home maker/Unemployed	111 96 57 20	39.1 33.8 20.1 7.0
**Mother tongue** Urdu Punjabi Sindhi Pushto Baluchi	207 29 22 21 5	72.4 10.2 7.7 7.4 1.8
**Family system** Joint Nuclear	175 109	61.6 38.4
**Living status** Alone With spouse only With children only Both spouse and children	34 57 134 59	12.0 20.0 47.2 20.8
**Source of income** Self Children Pension Savings Charity	114 76 55 34 5	40.1 26.8 19.4 12.0 1.8
**Income satisfaction** Yes No	203 81	71.5 28.5

**Figure 1:**
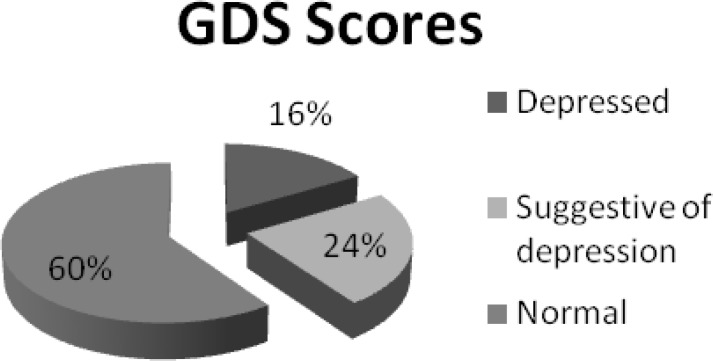
Geriatric Depression Scale (GDS) Scores of the community dwelling elderly in Karachi, Pakistan

Figure 1 highlighted the presence of depression among the 284 respondents. It was found that 47 (16.5%) respondents were depressed while 67 (23.6%) were suggestive of depression. Depression was mainly present in persons in the age range of 60-70 years.

When demographic variables were cross-tabulated with mental status, depression was more among men than in women (p<0.05). Similarly, depression was found to be statistically significant among married respondents (p<0.05) and those who were illiterate (p<0.001). Although a large proportion of the participants were satisfied with their income, this was found to be statistically significant (p<0.001) for depression among those who were not satisfied. Similarly, sleep was significantly disturbed (p<0.001) among the depressed participants. (See table 2).

## Discussion

An individual strives throughout his or her life for a peaceful old age, financial security, health, kinship and independence and yet these things remain elusive to many on this planet. The feelings of sadness that accompany routine life are usually appropriate and transitory, and can even present an opportunity for personal growth. However, when depression persists and impairs daily life, it may be an indication of a depressive disorder.

The present study reported 16.5% of the elderly population to be depressed whereas two hospital-based studies in Karachi showed depression in 23% and 20% among the elderly population (11,16). A systemic review revealed that a prevalence of 34% for anxiety and depressive disorders in Pakistani population (17). Moreover, there is a wide range (10% to 60%) of prevalence of depression in other locally published studies in Pakistan. These figures can be compared with neighboring country India having similar socio-demographic structure, the prevalence rates for depression showed a variation between 13% to 31% (18,19). A recent study done in Iran reported 23.5% of the subjects suffered from depression (20).

**Table 2:** Cross tabulation of demographic variables and mental status of community dwelling elderly in Karachi, Pakistan

**Variables**	**Mental Status**		**p-value** †
	**Normal ** **(%)**	**Suggestive of depression / Depressed (%)**	
**Sex**			
Male Female	116 (55.2) 51 (68.9)	94 (44.8) 23 (31.1)	0.04
**Age (in years)**			
60 – 70 71 – 80 > 80	113 (56.2) 44 (65.7) 10 (62.5)	88 (43.8) 23 (34.3) 6 (37.5)	NS
**Marital status**			
Single Married Widow Separated / Divorced	2 (16.7) 134 (61.5) 27 (62.8) 7 (63.6)	10 (83.3) 84 (38.5) 16 (37.2) 4 (36.4)	0.02
**Religion **			
Islam Others	150 (58.6) 20 (71.4)	63 (24.6) 4 (14.3)	NS
**Educational status**			
up to Intermediate up to Graduate/diploma Illiterate	81 (52.9) 43 (84.3) 46 (57.5)	41 (26.8) 6 (11.8) 20 (25)	< 0.001
**Occupational status**			
Working Retired/Housewife/ Unemployed	66 (58.6) 104 (59)	45 (41.4) 69 (41.0)	NS
**Mother tongue**			
Mohajir (Urdu speaking) Others	129 (62.3) 41 (53.2)	78 (37.7) 36 (46.8)	NS
**Family system**			
Joint Nuclear	106 (60.6) 64 (58.7)	69 (39.4) 45 (41.3)	NS
**Living status**			
Alone With spouse only With children only Both spouse and children	15 (44.1) 20 (50.9) 90 (62.2) 36 (61.0)	19 (55.9) 28 (49.1) 44 (32.8) 23 (39.0)	0.03
**Source of income**			
Self Any other means	64 (54.1) 106 (62.4)	50 (43.9) 64 (37.6)	NS
**Income satisfaction**			
Yes No	152 (74.9) 15 (18.5)	51 (25.1) 65 (81.5)	< 0.001
**Sleep disturbances**			
Yes No	60 (47.2) 107 (68.2)	67 (52.8) 50 (31.8)	< 0.001

**†**Variables were combined for statistical significance.

The relatively low prevalence of depression in this study can be attributed to the fact that the study was conducted in community dwelling elderly while previous studies conducted among elderly population of Karachi were based in a tertiary care setting. Moreover, elderly individuals coming to a tertiary care setting certainly present with health problems (the concept of wellness clinics is not widely prevalent in Pakistan) making them more prone to depression. In the community-dwelling individuals, normal elderly with controlled co-morbidities or no co-morbidities were also included from all socio-economic classes. Moreover, depression was mainly found in the 60-65 year age bracket indicating that a sizeable elderly population is in this age group. This is supported by UNICEF statistics (2009) which shows the average life expectancy of a Pakistani to 65 years (10).

In our study, depression was found to be mainly affecting males. A Brazilian study concluded that being male was a risk factor for depressive symptoms (21). On the other hand, studies have shown that women suffer from depression more than men do (16, 20, 22-24). There is a possibility that women seek treatment for depression while men are reluctant to accept their depressive state as they are the bread earners and are more prone to face the harsh realities of the world. However, in the present study, there were more male respondents than females (around ¾ of the sample). Further studies involving equal males and females can confirm this sex predilection.

In our study, those who were depressed, more individuals were residing in a joint family than in a nuclear family. Contrary to this, a Karachi based study showed that elderly living in a nuclear family system were 4.3 times more likely to suffer from depression than those living in a joint family system (11). This shift indicates that individuals residing in a joint family are exposed to new challenges, possibly of augmented generation gap and their inability to cope up with the ever-changing world. Conversely, individuals in a nuclear family do things their way and therefore escape the clashes that emerge when an experienced mind interact with the youthful, energetic, risk-taking young individual seeking pace with an elusive materialistic world. 

In the present study, 72% of the respondents were satisfied with their income. However, among those who were not satisfied, 82% were found to be depressed. These results were supported by other studies that showed significant differences between depression and poor financial status in elderly population (20,25). Hence, income satisfaction is central to depression in the elderly. Elderly, mostly retired find it extremely difficult to survive in financial uncertainty and rising costs of health care. A similar result was found in a study conducted in the US where found the incidence and persistence of depression to be higher among persons with low incomes (26). The unfulfilled economic needs bring frustration and certainly disappointment.

*Limitation*

The presence of depression warrants prompt intervention and treatment. Any positive scores above five on the GDS in the present study prompt an in-depth psychological assessment and evaluation for suicidality. However, this remains to be one of the limitations of the study due to the lack of follow-up by the researchers. 

*Conclusion*

The prevalence of geriatric depression reported by our study is less than that most of the other countries but still it is a significant percentage. If concerted and continuous efforts are endeavored by both health care professionals and the government, a reduction in the prevalence of geriatric depression is possible. As there are no specialized geriatric clinics in Pakistan, general physicians and other health care professionals need to be sensitized about geriatric depression, its risk factors and should treat it. Further, population-based studies should be carried out to estimate the prevalence of geriatric depression throughout Pakistan, explore risk factors and their relationship to depression and figure out potential solutions.

## Authors’ contributions

SMM conceived and designed the study, performed the statistical analysis, and helped to draft the manuscript and its critical final revision. DH carried out data collection, contributed in statistical analysis and drafted the study design evaluation and results. SNQ re-evaluated and interpreted the data, drafted and revised the manuscript. All authors read and approved the final manuscript before submission to the journal.

## Notes

**Declaration of interest: **None.
